# Assessment of the effects of Liuzijue Qigong on the lung function of COVID-19 patients during disease recovery: A protocol for systematic review and meta-analysis

**DOI:** 10.1097/MD.0000000000026866

**Published:** 2021-08-06

**Authors:** Jilin Wang, Yanru Cui, Shuang Liu, Jiangxue Zhou, Yingxia Sun

**Affiliations:** a College of Acupuncture and Massage, Shandong University of Traditional Chinese Medicine, Jinan, China; b First College of Clinical Medicine, Shandong University of Traditional Chinese Medicine, Jinan, Shandong, China.

**Keywords:** coronavirus disease 2019, Liuzijue Qigong, meta-analysis, protocol

## Abstract

**Background:**

The coronavirus disease 2019 (COVID-19) outbreak began in late 2019 and spread rapidly throughout China and then the rest of the world. COVID-19 is a serious respiratory disease and many patients’ exhibit varying levels of persistent parenchymal lung damage. However, there is currently a lack of effective rehabilitation treatments for COVID-19 patients with lung damage. Several clinical trials have shown that Liuzijue Qigong (LQG) can enhance the strength of respiratory muscles and overall quality of life. In this study, a meta-analysis approach was used to assess the effects of LQG on the lung function of COVID-19 patients during disease recovery.

**Methods:**

Eight databases will be explored for relevant investigations including China National Knowledge Infrastructure, Wanfang, VIP, China Biology Medicine, EMBASE, PubMed, Web of Science, and the Cochrane Library. All databases will be explored for articles published from inception through July 2021. Data will be extracted independently by 2 researchers according to the eligibility criteria. Finally, RevMan 5.3.0 will be implemented for statistical analyses.

**Results:**

The results of this study will show the effects of LQG on the lung function of COVID-19 patients during disease recovery and will be submitted to a peer-reviewed journal for publication.

**Conclusions:**

This study will provide reliable evidence based on the effects of LQG on the lung function of COVID-19 patients during disease recovery.

**Trial registration number:**

CRD42021268102

## 1 Introduction

The coronavirus disease 2019 (COVID-19) outbreak began in late 2019, and spread rapidly first through China and then throughout the globe,^[1,2]^ with the World Health Organization having stated a pandemic in March of 2020.^[3]^ COVID-19 patients experience a range of symptoms including cough, fever, lethargy, dyspnea, sputum production, and non-respiratory symptoms including myalgia, diarrhea, and headache.^[4,5]^ As of July 15, 2021, over 189,019,833 COVID-19 cases and 4,068,447 deaths have been recorded worldwide. As a serious respiratory disease, many COVID-19 patients exhibit varying levels of persistent parenchymal lung damage.^[6]^ It is thus vital that effort be made to identify safe and effective treatments that can aid in rehabilitative care for recovering COVID-19 patients.

The Chinese government has strongly advocated the integrative application of both Western medicines and traditional Chinese medicine (TCM) within the context of COVID-19 management in light of insights gained during the previous severe acute respiratory syndrome (SARS) outbreak.^[7]^ Liuzijue Qigong (LQG) is a traditional method of Chinese health physical activity that has long been practiced owing to its purported benefits to human health. LQG is performed through coordinated movement and exhalation while creating 6 specific sounds (xu, he, hu, si, chui, and xi). Many clinical trials have demonstrated the ability of LQG to ameliorate the standards of living and the strength of respiratory muscles.^[8–10]^ No systemic analyses of the impact of practicing LQG on pulmonary functions in cases that have been recovered from COVID-19 have been conducted to date, however. We, therefore, endeavor to execute a systematic review and meta-analysis of this topic to generate high-quality research evidence that can guide future clinical efforts to guide patient recovery.

## 2 Methods

### 2.1 Research registration

The registration of the current protocol has been performed via using the PROSPERO, registration number CRD42021268102, and will be executed in strict accordance with Preferred Reporting Items for Systematic Reviews and Meta-Analyses Protocols instructions.^[11]^

### 2.2 Eligibility criteria

#### 2.2.1 Research types

All randomized controlled trials reported in English or Chinese will be eligible for inclusion.

#### 2.2.2 Participant types

Eligible studies will be those evaluating patients in the recovery phase of a diagnosed COVID-19 infection without any age, gender, or ethnicity restrictions.

#### 2.2.3 Interventions and comparisons

Control patients will be those that have undergone routine treatment, whereas experimental group patients will be those that have undergone LQG therapy in addition to routine treatment.

#### 2.2.4 Findings

##### 2.2.4.1 Primary finding

Primary findings will include any rating scales that describe the patient pulmonary function.

##### 2.2.4.2 Secondary findings

1. Quality of life assessed by the 36-Item Short-Form Health Survey (SF-36).
2. Hamilton Anxiety Scale.

### 2.3 Search strategy

#### 2.3.1 Database search

In total, 8 databases will be explored for relevant investigations, containing 4 Chinese databanks (China National Knowledge Infrastructure, Wanfang Database, Chinese Science Journal Database [VIP], and China Biology Medicine) and 4 English databanks (EMBASE, PubMed, Web of Science, and Cochrane Library). All databases will be explored for articles published from inception through July 2021 using a combination of relevant search terms of MeSH terms. The PubMed search strategy is outlined in Table 1, and an identical approach will be employed for other databanks.

**Table 1:** Detailed search strategy in PubMed.

No.	Search terms
#1	COVID-19[MeSH Terms]
#2	SARS-CoV-2[MeSH Terms] OR 2019-nCoV [Title/Abstract] OR coronavirus disease 2019 [Title/Abstract] OR Novel coronavirus [Title/Abstract]
#3	#1 OR #2
#4	Qigong[MeSH Terms]
#5	Liuzijue [Title/Abstract] OR Liuzijue Qigong [Title/Abstract] OR Liuzijue exercise [Title/Abstract] OR Traditional Chinese Exercise [Title/Abstract] OR Six Syllable Formula [Title/Abstract]
#6	#4 OR #5
#7	Randomized Controlled Trial[Publication Type]
#8	Randomized Controlled Trial[Title/Abstract] OR Randomized [Title/Abstract] OR Placebo [Title/Abstract]
#9	#7 OR #8
#10	#3 AND #6 AND #9

#### 2.3.2 Searching other resources

Investigators will also seek to identify other relevant resources including unpublished trials in the Chinese Clinical Trial Registry and the US National Institutes of Health Ongoing Trials Register.

### 2.4 Study selection and data extraction

**Figure 1:**
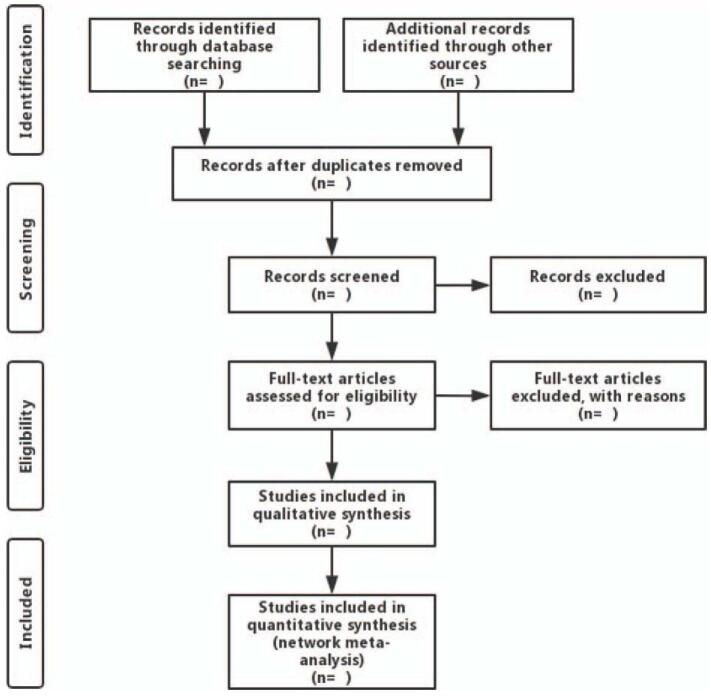
Flow diagram of literature retrieval.

Identified studies of potential relevance will initially be imported into Endnote X9, after which 2 investigators will independently screen the eligibility of these studies for inclusion in the final meta-analysis. Discords will be figured out by negotiation with a third researcher. Two researchers will then independently extract the following information from included investigations: author names, publication year, title, country, average age, gender, study design, participants, total case number, intervention measures, comparisons, outcomes, and other relevant details. When necessary information is unavailable, efforts will be made to contact the original authors. The study screening process is outlined in Figure 1.

### 2.5 Quality measurement

The Cochrane Risk of Bias Risk Assessment Tool will be independently employed by 2 researchers to evaluate study quality. The risk of bias will be assessed based on the following: random method, assignment concealment, approach to subject blinding, approach to result evaluation blinding, selective reporting, data integrity, and other biases. Each of these parameters will be assigned a high, low, or uncertain bias risk. Disagreements will be figured out by negotiation with a third investigator.

### 2.6 Statistical study

RevMan 5.3.0 will be implemented for statistical analyses. Heterogeneity among investigations will be detected using chi-squared tests. Fixed-effects models will be used to analyze data when no considerable heterogeneity is discerned (*P* ≥ .1 and *I*^2^ ≤ 50%), while random-effects models will otherwise be applied. Dichotomous data will be studied applying odds ratios with confidence intervals of around 95%, while continuous variables will be represented utilizing mean difference values and 95% confidence intervals.

### 2.7 Sensitivity analysis

When appropriate, sensitivity analyses will be accomplished by iteratively excluding individual studies from pooled analyses to assess result stability. When no qualitative changes in these outcomes are observed, the results will be considered stable.

### 2.8 Publication bias

To evaluate the potential for publication bias, the plots of the funnel will be drawn when sufficient studies were available (n ≥ 10). In addition, the risk of publication bias will be appraised by utilizing Egger assessment.

### 2.9 Evaluation of evidence quality

The quality of outcome-related evidence will be appraised by implementing the Grading of Recommendations Assessment, Development, and Evaluation method, and will be classified as either high, medium, low, or negligible.^[12]^

## 3 Discussion

Severe acute respiratory syndrome coronavirus 2 (SARS-CoV-2), the virus responsible for COVID-19, is almost tied to the SARS and Middle East respiratory syndrome coronavirus viruses responsible for prior outbreaks of severe pulmonary disease.^[13]^ SARS-CoV-2 infection can result in severe pneumonia-like symptoms in affected individuals,^[14]^ and a subset of COVID-19 survivors exhibit persistent physiological complications and radiological defects including abnormal lung functionality and restrictive ventilatory defects.^[15,16]^ Prior research has demonstrated that exercise training can facilitate pulmonary rehabilitation, improving symptoms such as dyspnea and the overall health of practitioners while reducing the need for other healthcare interventions. Beneficial outcomes have been ascribed to the use of TCM herbal remedies, Qigong exercises, and dietary advice formulated in accordance with TCM theories.^[17,18]^ Several clinical trials have generated evidence that LQG can enhance the strength of respiratory muscles and overall quality of life.^[8–10,19]^ At present, Shanghai University of Traditional Chinese Medicine recommends traditional LQG training as an approach to enhancing the restoration of normal pulmonary function in individuals recovering from COVID-19.^[20]^ To date, however, no meta-analyses have explored the influence of LQG on pulmonary outcomes in recovering COVID-19 patients. We will therefore conduct the meta-analysis proposed in this protocol to provide evidence-based guidance for aiding COVID-19 patient pulmonary rehabilitation.

## Author contributions

**Conceptualization:** Jilin Wang, Yanru Cui, Yingxia Sun.

**Data curation:** Jilin Wang, Yanru Cui, Shuang Liu, Jiangxue Zhou.

**Formal analysis:** Jilin Wang, Yanru Cui.

**Funding acquisition:** Yingxia Sun.

**Investigation:** Jilin Wang.

**Literature retrieval:** Yanru Cui, Shuang Liu, Jiangxue Zhou.

**Methodology:** Jilin Wang, Yanru Cui, Shuang Liu, Jiangxue Zhou, Yingxia Sun.

**Project administration:** Jilin Wang, Yingxia Sun.

**Resources:** Jilin Wang, Yanru Cui, Shuang Liu.

**Software:** Jilin Wang, Yanru Cui, Shuang Liu, Jiangxue Zhou.

**Supervision:** Yanru Cui, Yingxia Sun.

**Validation:** Jilin Wang.

**Visualization:** Jilin Wang.

**Writing – original draft:** Jilin Wang, Yanru Cui, Shuang Liu, Jiangxue Zhou, Yingxia Sun.

**Writing – review & editing:** Jilin Wang, Yanru Cui, Yingxia Sun.
